# Improved survival and tumor control with Interleukin-2 is associated with the development of immune-related adverse events: data from the PROCLAIM^SM^ registry

**DOI:** 10.1186/s40425-017-0307-5

**Published:** 2017-12-19

**Authors:** Brendan Curti, Gregory A. Daniels, David F. McDermott, Joseph I. Clark, Howard L. Kaufman, Theodore F. Logan, Jatinder Singh, Meenu Kaur, Theresa L. Luna, Nancy Gregory, Michael A. Morse, Michael K. K. Wong, Janice P. Dutcher

**Affiliations:** 1 Providence Portland Medical Center, 4805 NE Glisan Street, Portland, OR 97213 USA; 2 Moores Cancer Center, University of California San Diego, 9500 Gilman Drive, La Jolla, CA 92093 USA; 3 Beth Israel Deaconess Medical Center, 330 Brookline Avenue, Boston, MA 02215 USA; 4 Loyola University Medical Center, 2160 S First Avenue, Maywood, IL 60153 USA; 5 Rutgers Cancer Center Institute of New Jersey, 195 Little Albany Street, New Brunswick, NJ 08901 USA; 6 Indiana University Simon Cancer Center, 535 Barnhill Drive, Indianapolis, 46202 USA; 7 Primary Biostatistical Solutions, 2042 Carnarvon Ct, Victoria, BC V8R2V3 Canada; 8 Prometheus Laboratories, 9410 Carroll Park Drive, San Diego, CA 92121 USA; 9 Duke University Medical Center, 2301 Erwin Road, Durham, NC 27705 USA; 10 MD Anderson Cancer Center, 1515 Holcombe Blvd, Houston, TX 77030 USA; 11 Cancer Research Foundation of NY, 43 Longview Lane, Chappaqua, NY 10514 USA

**Keywords:** Immune-related adverse events, Interleukin-2, Renal cell carcinoma, Melanoma, Survival, PROCLAIM^SM^

## Abstract

**Background:**

Immune related adverse events (irAEs) are associated with immunotherapy for cancer and while results suggest improvement in tumor control and overall survival in those experiencing irAEs, the long-term impact is debated. We evaluated irAE reports related to high dose interleukin-2 therapy (IL-2) documented in the PROCLAIM^SM^ registry data base from 2008 to 2016 (NCT01415167, August 9, 2011).

**Methods:**

Reports on 1535 patients, including 623 with metastatic melanoma (mM) and 919 with metastatic renal cell cancer (mRCC) (7 patients had both diseases), were queried for irAEs. The timing of the event was categorized as occurring before, during or after IL-2 or related to any checkpoint inhibitor (CPI). mM patients and mRCC patients were analyzed separately. Tumor control [complete + partial response + stable disease (CR + PR + SD) was compared between those experiencing no irAE versus those with the development of irAEs. Survival was analyzed by tumor type related to timing of irAE and IL-2, and in those with or without exposure to CPI.

**Results:**

Median follow-up was 3.5+ years (range 1–8+ years), 152 irAEs were reported in 130 patients (8.4% of all PROCLAIM^SM^ patients): 99 (16%) in mM and 53 (5.8%) in mRCC patients. 31 irAEs occurred prior to IL-2, 24 during IL-2, and 97 after IL-2 therapy. 74 irAEs were attributed to IL-2 only (during/ after IL-2). Of the 97 post IL-2 irAEs, 24 were attributed to CPI, and 15 could not be distinguished as caused by IL-2 or CPI. Tumor control was 71% for those experiencing irAE, and 56% for those with no irAE (*p* = 0.0008). Overall survival was significantly greater for those experiencing irAEs during/ after IL-2 therapy, compared to those with no irAE or irAE before IL-2 therapy, in mM patients, median 48 months vs 18 months (*p* < 0.0001), and in mRCC patients, median 60 months vs 40 months (*p* = 0.0302), independent of CPI-related irAEs. IL-2-related irAEs were primarily vitiligo and thyroid dysfunction (70% of IL-2 related irAEs), with limited further impact.

**Conclusions:**

irAEs following IL-2 therapy are associated with improved tumor control and overall survival. IrAEs resulting from IL-2 and from CPIs are qualitatively different, and likely reflect different mechanisms of action of immune activation and response.

## Background

Immune-related adverse events (irAEs) are associated with immunotherapy for cancer. Data from the 1990’s reported irAEs following treatment with interferon-α, vaccines, activated lymphocytes, and interleukin-2 (IL-2), primarily in the treatment of the identified immune responsive cancers, melanoma and renal cell carcinoma [1–5]. In that early literature, the most common irAEs reported were thyroid dysfunction, both hypothyroidism and hyperthyroidism, with the frequent development of anti-thyroid antibodies [6–10]. Additional laboratory studies have described activated intra-thyroid lymphocytes [11]. Clinical observations have demonstrated frequent resolution of thyroid dysfunction without replacement therapy after completion of IL-2 treatment, although a number of patients do require thyroid replacement therapy, including maintenance replacement. Early reports observed thyroid dysfunction with immunotherapy in patients with either metastatic melanoma (mM) or metastatic renal cell cancer (mRCC), the most commonly treated tumor types. The correlation of the development of thyroid dysfunction and clinical outcome was controversial at that time [1–10]. The other most frequently reported irAE from IL-2 with or without interferon, specific to patients with mM, is vitiligo, a depigmentation phenomenon that requires no intervention. This has been associated with many types of immunotherapy evaluated in mM, including vaccines, interferon, IL-2, and adoptive cell transfer [12–15]. Rare serious irAEs have been reported with cytokine therapy, including IL-2, usually as case reports, and immune cell infiltrations were described in affected organs in some reports [16–19].

The acute toxicity noted with IL-2 administration has primarily been attributed to the induced capillary leak syndrome, as well as to effects of secondary cytokine release [20–27]. Early physiologic studies of IL-2 demonstrated the interaction of activated mononuclear cells with endothelium, causing leakage, and vascular accumulation of leukocytes and platelets impairing perfusion [20–23]. Also demonstrated were the effects of secondary cytokines on renal and hepatic function [24–27]. Clinically, the induced acute organ dysfunction (renal, hepatic, pulmonary) was and is observed during IL-2 administration and is rapidly reversible upon discontinuation of IL-2 [20].

The development of immune checkpoint inhibitors (CPIs) as anti-tumor therapeutic agents, directed at cytotoxic T-lymphocyte-associated antigen-4 receptor (CTLA-4) or programmed death receptor −1 (PD-1) or its ligand (PD-L1) has demonstrated the frequent occurrence and a broader spectrum of treatment-related irAEs. Some require acute therapeutic intervention and some require long-term management [28–32]. Data suggest the advent of irAEs may correlate with anti-tumor clinical benefit, although this has not been consistently seen [4, 7, 12, 14, 32, 33].

In Europe, IL-2 is administered at lower doses and for prolonged courses, therefore longer exposure, and irAEs are reported with these regimens as well [10]. Some have postulated that those who do well on such therapy will have longer exposure as the treatment continues, and therefore be at more risk of developing irAEs, rather than the irAE signaling better outcome [34]. However, a recent meta-analysis makes a strong case that the development of vitiligo following immunotherapy of any type has a positive prognostic impact in mM [14]. With regard to mRCC, reports are mixed, with some strongly indicating a relationship between irAEs and activation of the anti-tumor component of the immune response and others reporting no correlation [4, 7, 9, 10].

Therefore, to provide a contemporary evaluation of IL-2-related irAEs and clinical outcome, and to reflect treatment with high dose IL-2, administered as a short course, without maintenance, we searched the PROCLAIM^SM^ data-base (2006–2016), a registry study based on treatment with high dose IL-2 (Proleukin^R^). We report the spectrum of irAEs reported in the registry related to high dose IL-2, and evaluate their occurrence with respect to disease response and overall survival.

## Methods

### Patients (Table 1)

The PROCLAIM^SM^ registry is a data base study (NCT01415167) collecting information on patients treated with high dose IL-2 from 2006 and ongoing. The study was approved by the investigational review boards of the sites enrolling subjects and all patients provided written, informed consent. The data reported herein reflects patients treated from 2006 to 2016. As of the data cutoff, 1535 patients have been entered, including 426 accrued retrospectively, and 1109 (72%) accrued prospectively. This report includes information on 623 patients with mM and 919 patients with mRCC (7 patients had both diseases). These reports were queried for the development of irAEs. All reported AEs and SAEs were evaluated for potential irAEs, in addition to those reported directly as irAEs. AEs, SAEs, and irAEs were not audited, and reporting was a function of data entry at each participating site. Acute AEs and SAEs related to IL-2 administration which resolved after treatment were considered secondary to capillary leak syndrome or secondary cytokine effects, and consistent with standard management and assessments noted in previous reports [20–22, 35–38].

**Table 1:** Patients 2006–2016 (*n* = 1535)^a^

Metastatic Melanoma (mM)	623 patients
	62% male, 38% female
	Median age, 53 years,
	Range, 19–84 years
Metastatic Renal Cell Cancer (mRCC)	919 patients
	72% male, 28% female
	Median age, 57 years,
	Range, 18–84 years
7 patients had both mM and mRCC – counted in each subset	
Data retrospectively accrued	426 patients
Data prospectively accrued	1109 patients (72%)
Median follow-up	3.5+ years, range 1–8+ years

^a^7 patients had both diseases

The emphasis of PROCLAIM^SM^ is collection of data related to IL-2 treatment, however some reports included irAEs related to additional CPI treatment (not universally capturing all of these). IrAEs were categorized as occurring before, during or after IL-2 administration, and evaluated for relationship to IL-2 and/or to CPI administration. Median follow-up for the entire 1535 patients was 3.5+ years (range 1–8+ years).

### Statistical analysis

Overall response rate (ORR), complete + partial response (CR + PR) as well as tumor control (TC), CR + PR + stable disease (CR + PR + SD) was compared between all patients reported to have irAEs versus those with no irAE, using Fisher’s exact test. This was evaluated overall, and evaluated based on disease type. Overall survival (OS) of mM and mRCC patients was analyzed by disease type. OS curves were estimated by Kaplan-Meier method, and comparison was made between those with no-irAE or irAE occurring before IL-2 (group I) versus those with irAE occurring during or after IL-2 administration (group II), and significance was analyzed by the Log-rank test. Additional OS curves were constructed, limited to patients with irAEs related to IL-2 only, excluding those attributed to CPI.

## Results

One hundred fifty-two irAEs were reported in 130 patients (8.4% of all PROCLAIM^SM^ patients). This included 68% males and 32% females, with a median age of 55.5 years (range 24–75 years). Ninety-nine irAEs occurred in patients with mM (16% of mM patients), and 53 irAEs occurred in patients with mRCC (5.8% of mRCC patients). Thirty-one irAEs occurred in patients prior to their receiving IL-2 treatment, and some were attributed to prior therapy, such as adjuvant interferon. Twenty-four irAEs occurred during treatment with IL-2, and 97 irAEs developed after completion of IL-2 treatment. Seventy-four irAEs were attributed to IL-2 (occurring during or after IL-2). Twenty-four irAEs occurred after IL-2 and after subsequent CPI, and were attributed to CPI. With another 15 irAEs, the cause could not be distinguished between IL-2 or CPI (Table 2).

**Table 2:** Timing of Immune-Related Adverse Events with Relation to IL-2 Treatment

	irAEs/Total Patients (%)	Prior to IL-2	During IL-2	After IL-2
Total	152/1535 (8.4%)	31	24	97
mM	99/623 (16%)	16	12	71
mRCC	53/920 (5.8%)	15	12	26
Total irAEs		31	24	97
Related to IL-2		NA	During and after IL-2	74
Undetermined if Related to IL2 or CPI		NA		15
Related to CPI		NA		24

*mM* metastatic melanoma, *mRCC* metastatic renal cell cancer, *irAEs* immune-related adverse events, *IL-2* interleukin-2, *CPI* checkpoint inhibitor, *NA* - not applicable

We evaluated the median number of IL-2 doses received related to the development and timing of irAEs. The median number of doses for those with no irAEs was 19; for those with irAEs during IL-2 treatment (could be between courses, but total doses were counted) the median number of doses was 25, and for those who developed an irAE after IL-2, the median number of doses was 25. Therefore, there was no apparent dose-irAE relationship.

### Immune-related events (Table 3)

These data from PROCLAIM^SM^ confirm that the majority of IL-2 related irAEs were primarily vitiligo (all in patients with mM) and thyroid dysfunction (greater incidence in mRCC patients than mM patients) comprising 70% of irAEs attributed to IL-2, and with limited further impact on well-being. Less than 5% of limited irAEs related to IL-2 alone required intervention (i.e. holding IL-2), and these included joint pain, neuropathy, hepatitis. Serious irAEs related to IL-2 were reported and described below. Of note, in the PROCLAIM^SM^ data base, reporting of specific end-organ antibodies was not requested.

**Table 3:** Immune Related Adverse Events (irAEs) and Serious irAEs Reported

IrAEs related to Interleukin-2
1. 70% low level requiring no to minimal intervention
a. Vitiligo in mM patients
b. Thyroid dysfunction: 3 times more common in mRCC than mM
2. <5% irAEs reported requiring intervention (holding IL-2)
a. joint pain
b. Neuropathy
c. Hepatitis by enzyme elevation
15 Serious irAEs reported in overall Data Base among 152 irAEs^a^
6 in patients with mRCC – all during IL-2
9 in mM patients – 4 during IL-2; 5 after IL-2
9 patients - Myocarditis – all during IL-2
7 patients, only enzyme release; no clinical, electrocardiogram or echocardiogram findings
2 patients – elevated enzymes; 1- abnormal electrocardiogram and symptoms; 1 – elevated enzymes and asymptomatic ventricular arrhythmia.
1 patient each - Myasthenia Gravis, Guillain Barre - both related to IL-2
4 serious irAEs reported related to CPI^a^: 1 each: colitis, encephalopathy, neuropathy, uveitis

^a^irAEs related to CPI were not required to be reported

Additional irAEs were reported and associated with subsequent CPI treatment, but not all CPI treatment or CPI-related irAEs were captured within the PROCLAIM^SM^ data base. Examples of irAEs reported and attributed to CPI include auto-immune hemolytic anemia, vitiligo, psoriasis, colitis, hepatitis, thyroid dysfunction, uveitis, encephalopathy, adrenal insufficiency and hypophysitis. These data from PROCLAIM^SM^ confirm the frequent serious nature of irAEs related to CPI administration, often requiring acute intervention and possibly chronic management (52% of CPI irAEs – hypophysitis, colitis, hepatitis, uveitis).

### Serious immune-related events (Table 3)

Among patients experiencing irAEs and reported to the PROCLAIM^SM^ registry, there were 15 serious irAEs reported in 14 patients. Nine were in patients with mM and 6 were in patients with mRCC. Among the mRCC patients, all 6 serious irAEs occurred during IL-2 administration. Among the mM patients, 4 events were during IL-2 administration, and 5 were after IL-2. Of note, myocarditis was reported in 9 patients, all occurring during IL-2 administration and requiring termination of IL-2 therapy. This diagnosis was evaluated in all cases based on enzyme release (laboratory evaluation), and in 7, there were no clinical, ECG, or echocardiogram findings, consistent with IL-2-mediated myocarditis. In one case, there were abnormal ECG and echocardiogram findings and clinical symptoms, and in one case there were asymptomatic ventricular arrhythmias. All cases resolved, and there were no chronic effects noted. Two additional serious irAEs attributed to IL-2 were the development of myasthenia gravis in one patient and Guillain-Barre syndrome in one patient. Four additional serious irAEs reported in PROCLAIM^SM^ were attributed to subsequent CPI therapy, and include one each of colitis, encephalopathy, neuropathy, and uveitis.

### Response evaluation (Tables 4 and 5)

We evaluated best overall response (OR = CR + PR) and the achievement of tumor control (TC = CR + PR + SD) among all patients with irAEs, irrespective of timing of occurrence versus those with no irAEs. When evaluated overall, OR was observed in 34.5% of patients with irAEs and 21.8% of those with no irAEs, a significant differences in OR (*p* = 0.0014). TC was observed in 71% of patients experiencing irAEs compared with 56% of those who did not experience irAEs, a significant difference in tumor control (*p* = 0.0008).

**Table 4:** Tumor Response by Immune-Related Adverse Event Occurrence

Best Response^a^	irAE, *n* = 130 pts	No irAE, *n* = 1405 pts
Complete Response (CR)	8 (6.2%)	67 (4.8%)
Partial Response (PR)	37 (28.5%)	239 (17%)
Stable Disease (SD)	48 (37%)	483 (34.4%)
Progressive Disease	32 (25%)	470 (33.5%)
Missing	5 (3.8%)	146 (10.4%)
CR + PR + SD (n, %)	93 (71%)	789 (56%) ^b^p = 0.0008
CR + PR (n, %)	45 (34.6%)	306 (21.8%) ^c^p = 0.0014

*irAE* immune-related adverse event; pts.-patients;

^a^Best response is determined from responses captured between baseline IL-2 administration and prior to subsequent treatment

^b^Fisher’s exact test comparing CR + PR + SD by irAE to no irAE

^c^Fisher’s exact test comparing best response by irAE to no irAE

**Table 5:** Response by irAE and Disease Type

Best Response	mM		mRCC	
	irAE, *n* = 84	No irAE, *n* = 539	irAE, *n* = 46	No irAE, *n* = 873
Complete Response (CR)	5(6.0%)	23(4.3%)	3 (6.5%)	44(5.0%)
Partial Response (PR)	27 (32.1%)	89 (16.5%)	10 (21.7%)	153 (17.5%)
Stable Disease (SD)	29 (34.5%)	167 (31.0%)	19 (41.3%)	317 (36.3%)
Progressive Disease (PD)	19 (22.6%)	211 (39.1%)	13 (28.3%)	262 (30.0%)
Missing	4 (4.8%)	49 (9.1%)	1 (2.2%)	97 (11.1%)
CR + PR (n,%)	32 (38.1%)	112 (20.8%) ^a^P = 0.0058	13 (28.3%)	197 (22.6%) ^a^P = 0.5999
CR + PR+ SD (n, %)	61 (72.6%)	279 (51.8%) ^b^P = 0.0013	32 (69.6%)	514 (58.9%) ^b^P = 0.6263

^a^Fisher’s exact test comparing best response by irAE

^b^Fisher’s exact test comparing tumor control by irAE

We also evaluated TC and OR by disease type, among those with irAEs versus those with no irAEs. In patients with mM, OR was 38.1% in those with irAE and 20.8% in those with no irAE (*p* = 0.0058). TC was 72.6% in those with irAE and 51.8% in those with no irAE (*p* = 0.0013). In contrast, in patients with mRCC, OR was 28.3% in those with irAE and 22.6% in those with no irAE (*p* = 0.5999) and TC was 69.6% in those with irAE compared with 58.9% in those with no irAE (*p* = 0.6263), Thus, the significant difference observed in response and tumor control correlating with immune events is driven by the significant effect in patients with melanoma, although the numbers of mRCC patients is smaller.

### Overall survival

**Fig. 1:**
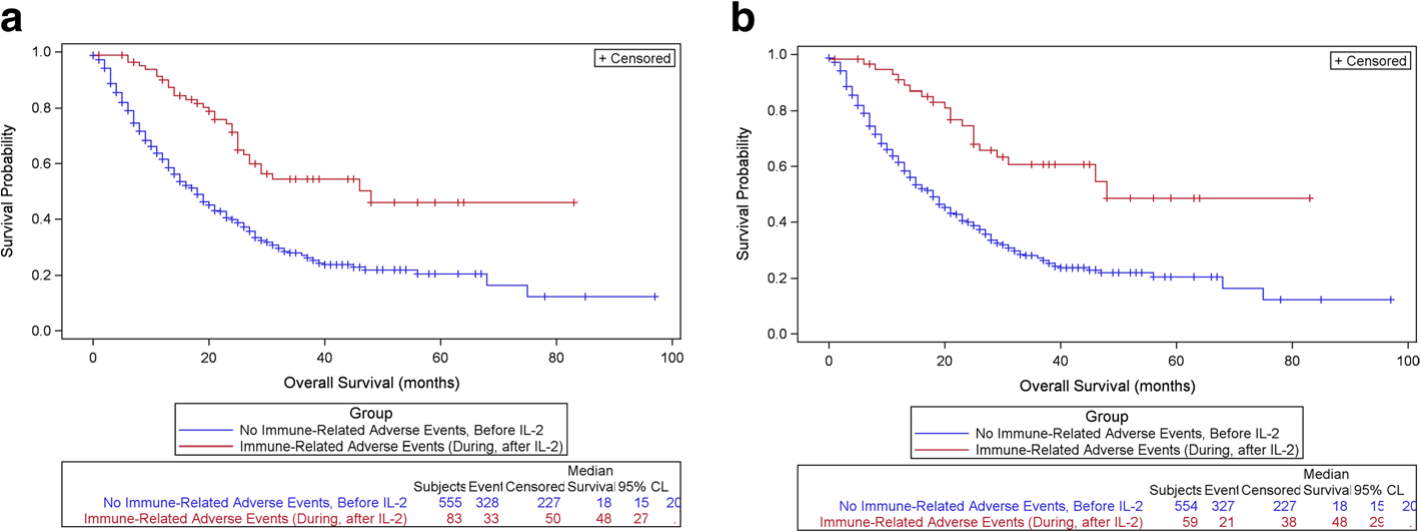
**a**: Overall Survival for Melanoma Patients with or without Immune-Related Adverse Events, Comparing Group I (no irAE/or irAE before IL-2) with Group II (irAE During/After IL-2). (Autoimmune Disease = irAE), *p* < 0.0001. **b**: Overall Survival in Patients with mM, comparing Group I with Group II, removing those with irAEs due to CPI. (Autoimmune Disease = irAE), *p* < 0.0001

**Fig. 2:**
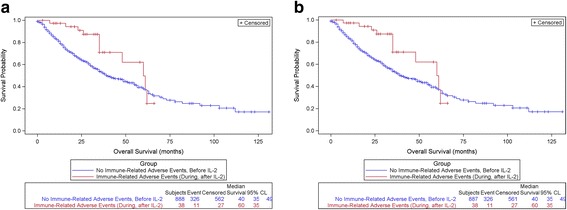
**a**: Overall Survival for mRCC Patients with or without Immune-Related Adverse Events, Comparing Group I (no irAE/or irAE before IL-2) with Group II (irAE During/After IL-2) (Autoimmune Disease = irAE), *p* = 0.0302. **b**: Overall Survival in Patients with mRCC, comparing Group I with Group II, removing those with irAE due to CPI. (Autoimmune Disease = irAE), *p* = 0.0302

OS was significantly greater among those experiencing irAEs during or after IL-2 treatment (Group II), compared with those with no irAEs or irAEs reported prior to IL-2 treatment (Group I). Among patients with mM, those experiencing irAE during/after IL-2 had a median survival of 48 months compared with 18 months for those with no or prior irAE (*p* < 0.0001), (Fig. 1a). Among patients with mRCC, those experiencing irAE during/after IL-2 had a median survival of 60 months compared with 40 months for those with no or prior irAE (*p* = 0.0302), (Fig. 2a). Subsequent analysis removed subjects whose irAEs were attributed to CPIs, and the OS curves remain statistically significantly greater for those experiencing irAEs during/after IL-2 and attributed to IL-2. (Figs. 1b, 2b).

## Discussion

Immunotherapy for cancer has long been associated with immune related adverse events (irAEs) affecting non-tumor target tissues [1–7]. The most frequently affected organ reported is the thyroid gland, with both hypo- and hyper dysfunction described. Other frequent end-organ targets are liver and skin, and also reactivation of prior autoimmune conditions, such as colitis, psoriasis, and rheumatoid arthritis. Although there is debate regarding the relationship of irAEs with respect to improved clinical outcome [4, 7, 9, 10, 12, 14], more recent data support this relationship [14, 33]. In this report, we have provided a contemporary evaluation of the relationship between IL-2 -related irAEs and clinical outcome of patients with mM and mRCC following high dose IL-2 treatment.

The PROCLAIM^SM^ data base provides largely prospectively accrued data on patients treated with high dose IL-2, and provides information on treatment course, toxicity, clinical outcome and potential long-term toxicity. Additionally, in this era of multiple new treatments, it provides an initial snapshot of outcome of sequential therapies for patients with mM and mRCC, and in some cases, sequential toxicities, based on the different treatments employed. This study however, may be limited by the lack of consistent reporting of low grade irAEs from either IL-2 or CPI, as well as no requirement for reporting of CPI use and subsequent emergence of CPI-related irAEs in these patients.

The majority of irAEs related to IL-2 therapy are low-grade, involving thyroid and skin, and not requiring major intervention or change in therapy [1–9, 14]. However, serious irAEs may occur and careful monitoring of patients receiving high dose IL-2 is always warranted, for both the acute toxicity of the treatment, and the potential for serious irAEs such as myocarditis and neurologic events [16, 18, 19]. Nevertheless, the vast majority of patients treated with high dose IL-2 have no long-term sequelae once treatment is complete. In the published literature, there is reported a broader spectrum and often greater intensity of irAEs noted in patients treated with CPI therapy [23, 33, 39]. Of note is the observation in patients treated with sequential immunotherapies, that there is not always recapitulation of the same toxicities (personal communications). It is hypothesized that differences in activation of T-cell responses and subsets are reflected in the different spectrum of irAEs observed among different classes of immunotherapy agents.

In this contemporary report of irAEs associated with high dose IL-2 treatment, the following observations have been made: [1] Overall, patients developing IL-2 related irAEs have significantly improved response (CR + PR) and tumor control (CR + PR + SD) compared to those who did not develop irAEs (Table 4). However, when analyzed by tumor type, this actually reflects a greater impact on response and tumor control in the melanoma patients experiencing irAEs, albeit the number of renal patients is smaller than the number of melanoma patients (Table 5). [2] The development of IL-2-related irAEs is associated with significantly improved overall survival in both mM and mRCC patients, when analyzed individually, compared to those with no irAEs (Figs. 1a and 2a), and excluding those with CPI-associated irAEs (Figs. 1b and 2b). These findings continue to support the association of immune activation from immunotherapy with better patient outcome. Of interest is the differential effect in mRCC, with a survival benefit, despite similar response rates in those with irAE and no irAE. This could be a factor of the smaller numbers, or in fact, to differences in effect on the disease itself. This may be relevant as immunotherapy expands into other disease entities.

These findings, and those reported with respect to CPIs also suggest new considerations as clinical trials move further into the study of combination immunotherapy for cancer. The combination of anti-CTLA-4 plus anti-PD-1 checkpoint inhibitors in patients with mM has demonstrated a higher response rate compared to single agent anti-CTLA-4, and an increase in grade 3/4 irAE rate [40–43].

However, a different combination reported by Prieto et al. of 3 phase II studies, two with the combination of anti-CTLA-4 and peptide vaccine and one with the combination of anti-CTLA-4 and high dose IL-2 in patients with advanced melanoma demonstrated combinability in terms of toxicity [44]. In the long-term follow-up of these studies, it is notable that the combination of anti-CTLA-4 with IL-2 demonstrated a doubling of complete response rate compared to the CR rate in the two studies with anti-CTLA-4 and peptide vaccine (17% vs 6%, 7%), and of the 15 CR patients, 14 were ongoing at the time of the report at 54+ to 99+ months [44]. Additionally, the median survival for the 3 regimens was greatest in those receiving anti-CTLA-4 with IL-2 (16 months vs 14 and 13 months) and the 5-year survival was 25% in the group with anti-CTLA-4 and IL-2. Of considerable interest was a reduced incidence of grade 3/4 irAEs among the patients receiving anti-CTLA-4 plus IL-2 compared to those in the two protocols receiving anti-CTLA-4 plus peptide vaccine (17% vs 29%, 32%). The authors postulate that IL-2-related induction of regulatory T-cells in addition to cytotoxic T-cells may decrease the activity of auto-reactive T-cells that may lead to serious irAEs and thus improve the therapeutic ratio [44]. This clearly needs further investigation.

The addition of activated tumor-infiltrating lymphocytes (TILs) in combination with IL-2, initially studied in advanced melanoma, but now in multiple tumor types, continues to be investigated. These approaches utilize shorter courses of high dose IL-2 or different dose/schedules of IL-2 [45, 46]. Single institution studies report greater responses and apparent improved survival [45, 46]. Historical series have only reported acute toxicities, and not irAEs. Numerous trials are ongoing, including multicenter trials, with central production of TILs, as well others exploring differing doses of IL-2, and combinations of IL-2, TILs and CPIs. The occurrence of irAEs, either early or late should be compiled.

Another approach to enhancing the immunotherapeutic effect of IL-2 involves the combination of localized radiation (producing tumor antigen release) followed by high dose IL-2. A recent publication by Hannan et al. has demonstrated the potential for synergy from this approach in patients with mRCC [47]. In this single institution report, there appeared to be enhanced clinical activity and follow-up is ongoing. Of interest would be the level of low grade versus high grade irAEs with this approach. The investigators do not note unexpected toxicity (Hannan, personal communication) Others are exploring this combination also, among patients with both mM and mRCC.

Considering the recent evaluation of the combination of anti-CTLA4 plus anti-PD1 in mM and the similarity in outcome with anti-PD1 alone, as well as the less severe irAE profile with anti- PD1, this agent may in fact be a clinically very feasible agent to combine with IL-2. Studies are ongoing. The results from the Prieto, et al. report support that serious irAEs from the combination of a checkpoint inhibitor (anti-CTLA-4) and IL-2 will not a priori be additive, and in fact might be less, and manageable with such combinations [44]. They did not report irAEs of less than grade 3 severity, which in our series reflect 70% of IL-2 related irAEs. A recent review of the newer immunotherapies is entitled “autoimmune events - the Achilles’ heel of immunotherapy” [39], but it is also possible that different combinations will lead to different spectra of toxicity, with the potential for enhanced durable response and survival and lower grade irAEs.

The advantages of an immunotherapeutic agent such as high dose IL-2 for consideration in combination therapy are the following: a) the short course of therapy, b) the extensive experience with its administration, c) the very limited extent of long-term irAE complications and importantly, d) the documented long-term disease-free survivorship demonstrated over many decades [44, 48–54]. Therefore, attempts to enhance antigen presentation, or selective cytotoxic T-cell activation, or ex vivo T-cell activation, among other approaches, should capitalize on this investigation of combined immunotherapy.

## Conclusions

In summary, the irAE profile observed with high dose IL-2 in this registry study demonstrates generally manageable events, and rare long-term toxicity. There does appear to be a correlation between irAE development and improved response (mM) and survival (mM and mRCC) following treatment with high dose IL-2. High dose IL-2 is a short, manageable treatment course that may be combinable with the newer CPI agents and other developing immunotherapies, and these combinations will not necessarily lead to additive irAEs and toxicity. Obviously, the ultimate goal is to enhance response rate, tumor control and overall survival.

## Abbreviations

AE: Adverse events
CPI: Checkpoint inhibitor
CR: Complete response
CTLA-4: Cytotoxic T-lymphocyte-associated antigen-4 receptor
IL-2: Interleukin-2
irAEs: Immune-related adverse events
mM: Metastatic melanoma
mRCC: Metastatic renal cell carcinoma
n: Number
OS: Overall survival
PD-1: Programmed death receptor-1
PD-L1: Programmed death receptor-ligand-1
PR: Partial response
SAE: Serious adverse event
SD: Stable disease
TC: Tumor control
TILs: Tumor infiltrating lymphocytes
